# Opening the black box of non-pharmacological care in systemic sclerosis: a cross-sectional online survey of Dutch health professionals

**DOI:** 10.1007/s00296-020-04765-4

**Published:** 2020-12-23

**Authors:** Juliane K. Stöcker, Madelon C. Vonk, Frank H. J. van den Hoogen, Maria W. G. Nijhuis-van der Sanden, Julia Spierings, J. Bart Staal, Ton Satink, Cornelia H. M. van den Ende, The ARCH study group, C. W. Y. Appels, R. Bakker, S. Bazen, F. Bender, H. Berkers, A. E. van der Bijl, E. W. F. Blaas, F. Bonte-Mineur, H. Boerhof, G. W. M. Boerrigter, R. Bos, W. J. W. Bos, C. Bruggink, P. D. M. de Buck, E. van de Burgt, F. S. Catarinella, S. Denktaş, M. Derksen, T. Dhondai, M. Eimers, R. J. Goekoop, E. van Gorp, L. D. M. Gossens, A. Groenendaal-Boender, R. van der Heijden, M. J. Herenius, J. Hofman, J. Huizer, M. Hulsinga, B. Jilderda, R. Jonkers, S. J. de Jong, C. G. M. Kallenberg, M. A. E. de Kanter, M. van Klei, H. Knaapen-Hans, H. Knaken, I. Koopmans, P. Krop, L. Kwakkenbos, R. Langenhuijsen, S. Leroux, I. C. Lether, M. Limper, M. Lohr, L. van Mourik, D. J. Mulder, A. Muns, S. Otter, S. Persijn, W. F. Peter, J. Potjewijd, B. Rave, H. de Ruiter, C. G. Schoemaker, T. H. M. Schoonbrood, A. A. Schouffoer, M. Soeters, A. J. Stel, J. Stöcker, Y. K. O. Teng, E. Ton, A. van Veen, S. Verhaar, P. M. J. Verhoeven, E. Voogt, M. Voortman, Y. de Vries, A. E. Voskuyl, J. E. M. Wijbrans-Roodbergen

**Affiliations:** 1 Department of Rheumatology, Sint Maartenskliniek, P.O. Box 9011, 6500 GM Nijmegen, The Netherlands; 2 Musculoskeletal Rehabilitation Research Group, HAN University of Applied Sciences, Nijmegen, The Netherlands; 3 Department of Rheumatology, Radboud University Medical Center, Nijmegen, The Netherlands; 4 Radboud Institute for Health Sciences, IQ Healthcare, Radboud University Medical Center, Nijmegen, The Netherlands; 5 Department of Rheumatology and Clinical Immunology, University Medical Center Utrecht, Utrecht, The Netherlands; 6 Research Group Neuro Rehabilitation, HAN University of Applied Sciences, Nijmegen, The Netherlands; 7 European Masters of Science in Occupational Therapy, Amsterdam, The Netherlands

**Keywords:** Systemic sclerosis, Health professionals, Unmet care needs, Cross sectional survey

## Abstract

The objective is to describe the spectrum of the health professional (HP) treatment approach for systemic sclerosis (SSc) from the perspective of Dutch HPs, including alignment of treatment goals set by HPs with self-reported referral reasons, coverage of patient-reported unmet care needs, and quality of communication between HPs and rheumatologists. Dutch HPs were invited through their patients with SSc to complete an anonymous online survey. The survey covered referral reasons, treatment goals, and interventions of the last patient treated, as well as the perceived quality of communication between HPs and rheumatologists. Referral reasons and treatment targets were linked to the International Classification of Functioning, Disability and Health following the refined ICF Linking Rules. Seventy-nine HPs from 8 professions (including 58 physiotherapists, 73%) completed the survey. One hundred and thirty-three different referral reasons were reported, yielding 58 different ICF codes, with 41 (70.7%) being linked to the ICF domain “body structures and functions.” The reported interventions focused on body functions/structures (27.9%), training of daily activities (25.6%), education and advice (26.3%), and psychosocial interventions (20.2%). The quality of communication between HPs and rheumatologists was perceived as low. Our findings revealed numerous treatment options offered by Dutch HPs addressing the unmet care needs of patients with SSc. There is an overlap in the content of the various HP disciplines, and HP treatment goals are not sufficiently aligned with referrals of rheumatologists. HP treatment offer seemed inefficiently organized, possibly precluding rheumatologists from making targeted referrals. Communication between rheumatologists and HPs should be improved.

## Background

Systemic sclerosis (SSc) is a rare and complex autoimmune disease with large differences in severity and extent. Its worldwide incidence is an estimated 13 people per million per year, and its prevalence is approximately 200 people per million [1]. SSc has a heterogeneous and often progressive nature that involves skin, vessels, joints, and internal organs, and it significantly impairs patients’ daily functioning and quality of life [2, 3]. There is no effective treatment or cure for SSc yet, meaning that treatment is primarily aimed at controlling symptoms and maintaining quality of life [4]. As treatment options for life-threatening, organ-based complications improve, treatment approaches for nonfatal SSc complications require increased attention [5–7].

Due to the direct impact of SSc on daily functioning and psychosocial well-being of patients, non-pharmacological management and treatments are a key element of SSc care [8]. Health professionals in rheumatology (HPs), including occupational therapists, physiotherapists, psychologists, and social workers, play a vital role in the support of individuals with SSc manage their nonfatal SSc complications [9]. So far, no recommendations for the non-pharmacological care for SSc are formulated, but several high-quality randomized trials support the use of non-pharmacological treatment options to reduce the clinical burden of a variety of symptoms [6, 10, 11]. In addition, care by health professionals is also based on treatments proven to be effective in other rheumatic diseases. For instance, promising approaches to address fatigue in patients with RA and SLE are also applicable for patients with SSc [12–14].

In the past decades, owing to changes in the Dutch health care system, HP treatment has been transferred from hospital-based team care to a primary care setting. As a result, patients with SSc have more often been referred to HPs working in monodisciplinary primary care settings. Considering that, rheumatologists have more confidence in HP colleagues with whom they work on a daily basis in the same institution [15]. This development may have negatively affected rheumatologists’ knowledge of HP treatment options, adequate coordination of treatment, and the quality of communication between rheumatologists and HPs.

SSc patients consider non-pharmacological care as one of the five main issues affecting the quality of SSc care in need of improvement [9, 16, 17]. Spierings et al. identified the following the top five unmet care needs of patients with SSc: fatigue, Raynaud’s phenomenon, physical limitations, and impaired hand and joint function [17]. It remains unknown to what extent these five unmet care needs are addressed by HPs in the treatment of patients with SSc.

Therefore, the aim of our study was to examine the content and alignment of care delivered by Dutch HPs with patients’ most important needs.

## Methods

### Design

A cross-sectional study using a web-based survey (SurveyMonkey^®^.com) was conducted to make an inventory of perceptions of Dutch HP treating patients with SSc. This study was reported according to the Strengthening the Reporting of Observational Studies in Epidemiology (STROBE) and using the Checklist for Reporting Results of Internet E-Surveys (CHERRIES) [18, 19].

### Survey

The survey questions were based on several preliminary investigations conducted by the Arthritis Research and Collaboration Hub (ARCH) working group: a literature review, three semi-structured multicenter focus group interviews, and individual interviews among patients, HPs, and rheumatologists [17].

The 23 survey questions were distributed over 14 webpages and divided into 4 domains: socio-demographic and work setting-related characteristics (12), referral to non-pharmacological care (2), treatment (5), and perceived quality of communication (4). The survey included both open-ended questions, asking the participants to answer in their own words, and closed questions, providing multiple-choice and multiple-response questions.

#### Socio-demographic and work setting-related questions

The survey started with 12 socio-demographic and work setting-related questions: sex (woman, man); age (free text); educational level (bachelor, master, Ph.D., and others); work experience (free text); profession (dietitian, occupational therapist, physiotherapist, hand therapist, speech therapists, social worker, dental hygienist, exercise therapist, podiatrist, psychologist, and others); caseload of SSc patients in the past year (0–2, 3–6, 7 or more); SSc specialization (yes/no); working hours per week (32 or more, 20–31, 12–19, others); work domains of the past 5 years (patient care, research, education, management, and others); current work setting (academic hospital, regional hospital, health center, private practice, nursing home, rehabilitation center, and others); SSc specialization of work setting (yes, no); and participation in multidisciplinary SSc consultations (yes, no, and others).

#### Referral to non-pharmacological care

Types of referrer were assessed by means of a list of seven medical disciplines and an option to add new items. With the following answering format: never, sometimes, and always. Most common reasons for SSc referrals, as reported by HPs, were assessed by an open-ended question with three options for free text responses.

#### Treatment

Five questions assessed the HP treatments. HPs were asked to consider the last SSc patient treated to assess the following items: type of SSc (limited SSc, diffuse SSc, I do not know, others), main treatment goals (open-ended question with 3 options for free text responses), main interventions (multiresponse question divided into 4 domains, body structure and functions (20 items), activity and participation (9 items), education and advice (20 items), and psychosocial interventions (12 items). These multiresponse questions were used to prioritize items, participants could choose a maximum of three options, including an option to add a new item. Duration of HP treatment was assessed with two free text questions: duration in weeks and number of treatment contacts.

#### Perceived quality of communication

Perceived quality of communication was assessed by adapting four items of the Dutch version of the Consumer Quality Index Rheumatoid Arthritis (CQI-RA) (version 2.0), subscale ‘Communication’. The CQI-RA was found to be a reliable measure for patients’ experiences with the quality of rheumatic care [20]. For our study, we used the items ‘Parallel treatments were adjusted to one another’, ‘Various advises were integrated’, ‘Caregivers kept their appointments’ and ‘Caregivers were aware of other activities of caregivers’ and adapted them to measure the experiences of HP (see Table 4). The answering format of the items was: never, sometimes, usually, and always.

The survey was evaluated by members of the ARCH SSc working group and a patient panel of five patients. Only an individual code and Internet Protocol (IP) address was registered to guarantee the anonymity of the participants. Pilot testing of the questionnaire was undertaken in five HPs to ensure the relevance of the questions [17].

### Sampling strategy

Sampling followed a targeted snowball sampling strategy [21]. Dutch HPs from different disciplines (including physiotherapists, occupational therapists, podiatrists, hand therapists, dietitians, dental hygienists, speech therapists, psychologists, and social workers) were eligible to participate in the study, if they were currently treating or had previously treated patients with SSc. There were no participation restrictions on the workplace, the case load, or the working environment. HPs were invited by their own patients with SSc who participated in a large-scale survey among 650 Dutch patients with SSc set up by the ARCH working group. Patients with SSc who participated in the study were asked to ‘snowball’ their treating HPs by providing them with an internet link we offered, or by writing down the name and address of the workplace of the HP, enabling us to invite the HP to take part in the study. An estimation of the sample size was not possible due to snowballing as sampling strategy and the unknown number of HPs working with SSc patients in the Netherlands. Eligible participants had 4 months to voluntarily complete the survey (December 2017 to March 2018). The survey link was open from the time the participants were first informed about the study. The cover letter, displayed on the first page of the survey, provided details about the background and purpose of the survey, along with the estimated duration of the survey (15 min). Informed consent was taken at the beginning of the survey.

### Data handling and confidentiality

IP address checks have been performed to avoid duplicate answers from one respondent. The data processing was completely anonymous, with the IP addresses remaining with the first and corresponding author. A second author (CHME) had access to the individual codes and synthesized data without associated IDs. Only completed surveys were included in the analyses.

Ethical approval was obtained from the Institutional Review Board of the Radboudumc Nijmegen, the Netherlands, protocol (2017: 3621).

### Data analysis

#### Statistical analysis

Socio-demographic and work setting-related data, HP interventions and perceived quality of communication were analyzed descriptively. Continuous variables, following a normal distribution, were reported as means and SD and categorical variables as absolute numbers and percentages. Statistical analyses were conducted using Stata/IC 13.1 (StataCorp LP, College Station, TX).

#### Analysis of open-ended questions

The qualitative data analysis of the answers to open-ended questions followed an adapted form of “meaning condensation” [22]. First, all answers to the open questions about referral reasons and treatment goals were read through by the principal investigator (JS) to obtain an overview of the collected data. Second, all data were divided in ‘meaning units’, defined as specific text units, either a few words or a part of a sentence with a common meaning. Third, concepts within each meaning unit were identified. Sometimes one meaning unit could contain several concepts. For instance, the meaning unit, “Staying fit so that my client can keep walking > 5 km.” contains the concepts ‘maintaining physical fitness’ and ‘walking longer distances’. All resulting concepts were linked to the most appropriate ICF category according to established linking rules [23, 24]. The purpose of the matching process was to translate the concepts from the HPs’ answers into the most appropriate ICF categories. The ICF classification uses a hierarchical structure organized in chapters, or ‘first level’ categories, which subdivide the four separate concepts of body functions, Body structures, activities and participation and environmental factors. Each chapter contains numerous categories (second, third, and fourth levels), which form the classification unit. The specificity increases from the first to the fourth level. As an example, the concept ‘walking longer distances’ was linked to *d450 Walking.* ‘Maintaining physical fitness’ was linked to *d5701 Managing diet and fitness*.

In accordance with the linking rules, interactive discussions were held to resolve coding discrepancies (JS and CHME). Finally, all assigned ICF codes were re-read repeatedly by the main coder (JS) to ensure that the linked ICF codes reflected the meaningfulness of the concept.

Through this process, the large number of answers to the open questions on referral reasons and treatment goals were reduced to a smaller amount of clearly defined ICF terms. These were used to compare treatment goals with reasons for referral and unmet care needs.

## Results

### Participants, origin, and content of referrals

We obtained 81 completed surveys. One duplicate response set was identified and excluded from the analysis, and another set was excluded for not meeting the inclusion criteria; the person was a medical doctor. Thus, data from 79 surveys taken by eight HP professions were analyzed. Table 1 presents the HPs’ socio-demographic and work-related characteristics.

**Table 1:** Characteristics of 79 health professionals working with patients with SSc and frequency of referrals from different sources

Characteristics	
Female, *n* (%)	52 (65.8)
Age, years; mean (SD), range	41.2 (13.6), 22–82
Education level, *n* (%)	
Bachelor diploma	53 (67.1)
Master diploma	22 (27.9)
PhD	4 (5,1)
Patients with SSc per year, *n* (%)	
0–2 patients	60 (76.0)
3–6 patients	14 (17.7)
≥ 7 patients	5 (6.3)
Specialized in SSc treatment *n* (%)	21 (26.6)
Institution/practice specialized in SSc treatment *n* (%)	11 (13.9)
Regular participation in multidisciplinary consultation of patients with SSc *n* (%)	6 (5.6)
Profession *n* (%)	
Physiotherapist	58 (73.5)
Dietitian	6 (7.6)
Occupational therapist	5 (6.3)
Podiatrist	4 (5,1)
Skin therapist	3 (3.8)
Speech and language therapist	1 (1.3)
Dental hygienist	1 (1.3)
Psychologist	1 (1.3)
Practice setting *n* (%)*	
Private practice	60 (69.0)
Hospital or treatment center	27 (31.1)
School/university	2 (2.3)
Other	7 (8.1)
Category of work during the last 5 years *n* (%)*	
Clinical patient care/rehabilitation	83 (73.6)
Education	13 (11.6)
Management	12 (10.7)
Research	8 (7.1)
Years worked in clinical practice as a health professional, years; mean (SD), range	16.9 (12.2), 0.5–42.0

Frequency of HP referrals from different sources *n* (%)*,**	Never	Some	Most/all
General practitioner	62 (81.6)	13 (17.1)	1 (1.3)
Rheumatologist	20 (26.3)	41 (54.0)	15 (19.7)
Dermatologist	72 (94.7)	3 (4.0)	1 (1.3)
Other medical specialist	62 (81.6)	11 (14.5)	3 (4.0)
Other health professional	72 (94.7)	4 (5.3)	0 (0.0)
Self-referral	54 (71.1)	17 (22.4)	5 (6.6)

*Multiple answers possible

**n = 76

The larger proportion of participants was female (*n* = 52; 67%). Physiotherapists were the largest group represented (*n* = 58; 73%), followed by dietitians (*n* = 6; 8%) and occupational therapists (*n* = 5; 6%). Nineteen (24%) of the respondents reported to have treated 3 or more patients with SSc in the past year. Most HPs (*n* = 60, 69%) worked in private practices. In all, 21 (26.6%) HPs felt specialized in SSc care, and 11 (13.9%) found that their workplace was specialized in SSc. Only six HPs (5.6%) regularly participated in multidisciplinary SSc meetings.

HPs reported that rheumatologists were the most frequent referrers (*n* = 56, 73.7%). Nearly one-third (*n* = 22, 29.0%) of the reported referrals were patient self-referrals. All other referrals were distributed among general practitioners (*n* = 14, 18.4%), dermatologists (*n* = 4, 5.3%), other medical specialists (*n* = 14, 18.5%) and other HPs (*n* = 4, 5.3%).

The 129 concepts on referral reasons, collected from open-ended questions, could be linked to 47 unique ICF codes and included 31 ICF codes on Body structures and functions (89 concepts), 13 on Activities and participation (36 concepts), and 3 on Environmental factors (4 concepts). Table 2 presents the ten most frequently mentioned referral reasons together with the reporting disciplines. Seven of the ten most frequently cited referral reasons were aimed at Body structures and functions. In addition, up to four HP disciplines received referrals with identical referral reasons.

**Table 2:** Ten most frequently mentioned referral reasons and reporting disciplines

	ICF code		*n*	*n* per HP discipline* reporting the referral reason
1	Aerobic capacity	b4551	11	11 PT
2	Sensation of pain	b280	8	8 PT
3	Mobility of joint functions	b710	8	6 PT, 1 ST, 1 P
4	Carrying out daily routine	d230	8	6 PT, 2 OT
5	Respiration functions	b440	7	7 PT
6	Managing one’s own activity level	d2303	7	4 PT, 3 OT
7	Energy level	b1300	6	3 PT, 1 OT, 1 D, 1 P
8	Weight maintenance functions	b530	6	6 D
9	Muscle power functions	b730	5	5 PT
10	Hand and arm use	d445	5	1 PT, 1 HT, 3 ET

*PT physiotherapist, *ST* skin therapist, *P* podiatrist, *OT* occupational therapist, *D* dietitian

### Treatment goals and interventions

Analysis of the reported treatment goals revealed 209 concepts that could be coded into 66 unique ICF codes. Most of the treatment goals were aimed at Body structures and functions (*n* = 35 ICF codes, consisting of 119 concepts), a smaller part focused on Activities and participation (*n* = 27 ICF codes, consisting of 86 concepts) and only a small amount of the treatment goals aimed at Environmental factors (*n* = 4 ICF codes, consisting of four concepts). Nine participants did not report any treatment goals. Table 3 shows the ten most frequently mentioned treatment goals, together with the number of disciplines that reported the respective treatment goal.

**Table 3:** Ten most frequently mentioned treatment goals and reporting disciplines

	ICF code		*n*	*n* per HP discipline* reporting the referral reason
1	Aerobic capacity	b4551	25	25 PT
2	Managing daily routine	d2301	15	13 PT, 2 OT
3	Managing one’s own activity level	d2303	15	12 PT, 3 OT
4	Mobility of several joints	b7101	12	11 PT, 1 P
5	Muscle power functions	b730	12	12 PT
6	Sensation of pain	b280	11	9 PT, 2 P
7	Managing diet and fitness	d5701	9	4 PT, 5 D
8	Other functions of the skin	b830	7	6 PT, 1 ST
9	Moving around	d455	6	6 PT
10	Hand and arm use	d445	6	6 PT

*PT physiotherapist, *ST* skin therapist, *P* podiatrist, *OT* occupational therapist, *D* dietitian

A total of 605 interventions (8.8 average per participant) were reported, with the treatment focus more or less evenly distributed across the following 4 components: Bodily functions/structures (27.9%), Training of activities (25.6%), Education/advice/instruction (26.3%), and Psychosocial interventions (20.2%). The most frequently mentioned interventions (top four per topic) are presented in Table 4. Within these most frequently mentioned interventions, we found five interventions or strategies that are applied by up to six different HP disciplines: walking/cycling (4), exercise activities/sport (6), household (5), self-management/self-monitoring (4), and motivational interviewing (5) (Table 4).

**Table 4:** Interventions applied by the 79 HPs, top 4 per topic

Intervention	*n* (%)	Number of HP per discipline* focusing on the intervention (disciplines *n*)
Body functions and/or structures		
Physical activity promotion	38 (48.1)	33 PT, 1 OT, 4 D
Training of body functions (e.g., muscular strength, range of motion)	34 (43.0)	34 PT
Aerobic capacity training	25 (31.7)	25 PT
Balance/coordination training	14 (17.7)	13 PT, 1 P
Activities		
Walking/biking	45 (57.0)	39 PT, 3 D, 2 P, 1 S (4)
Movement activities/sports	45 (57.0)	37 PT, 3 D, 2 HT, 1 S, 1 P, 1 OT (6)
Leisure activities	19 (24.1)	17 PT, 1 ET, 1 ST
Household	16 (20.3)	11 PT, 2 OT, 1 HT, 1 D, 1 P (5)
Education/advice/instruction		
Graded activity	44 (55.7)	38 PT, 4 OT, 1 HT, 1 P (4)
Physical activity	42 (53.2)	39 PT, 1 D, 2 HT
Lifestyle (e.g., smoking, cold, silver gloves)	14 (17.7)	12 PT, 2 OT
Energy conservation	12 (15.2)	10 PT, 1 OT, 1 D
Psychosocial interventions		
Self-management/self-monitoring	53 (67.1)	42 PT, 4 OT, 3 D, 2P, 2 KT (5)
Relaxation strategies/stress management/biofeedback therapy	23 (29.1)	20 PT, 2 OT, 1 D
Motivational Interviewing	13 (16.5)	6 PT, 3 OT, 2 D, 1 DH, 1 P (5)
Problem-solving training	10 (12.7)	9 PT, 1 S

*PT physiotherapist, *P* podiatrist, *OT* occupational therapist, *D* dietitian, *HT* hand therapist, *DH* dental hygienist, *S* speech therapist, *ST* skin therapist

### Alignment of referral reasons and HP treatment goals

In all, 17 of 129 (13.2%) referral reason concepts matched with one of the treatment goal concepts at the patient level. In 10 cases, referral reasons fully matched with treatment goals. The ICF codes d230/2303 (Carrying out daily routine/Managing one’s own activity level) corresponded in four cases, whereas b4551 (Aerobic capacity), and b280 (Sensation of pain) corresponded in two cases. The other corresponding codes were: b4550/b4551 (General physical endurance/Aerobic capacity), b710/b7101 (Mobility of joint functions/Mobility of several joints), b730 (Muscle power functions), d445 (Hand and arm use), s320/s3200 (Structure of mouth/Teeth), and s7502 (Structure of ankle and foot).

### Correspondence between treatment goals and unmet care needs

The examined unmet care needs *fatigue, Raynaud’s phenomenon, joint problems, physical function, and hand function* were covered by 108 out of the total of 209 ICF codes extracted from reported HP treatment goals. Since the unmet care needs described relate to physical symptoms and not to the transcending health information such as situations and daily activities, almost exclusively ICF codes from chapters b (Body functions) and s (Body structures) could be assigned.

In 57 (81.4%) of the 70 cases in which participants provided information about treatment goals, we found ICF codes directly associated with 1 or more of the 5 unmet care needs. In half of all cases, we found agreement with the unmet care need *fatigue* (*n* = 16, 22.9%), *Raynaud’s phenomenon* (*n* = 12, 17.1%), *joint problems* (*n* = 18, 25.7%), *physical function* (*n* = 35, 50.0%), and *hand function* with nine associable cases (12.9%). In 13 cases, we did not find a direct connection with 1 of the 5 unmet care needs; 7 of them concerned the participating dieticians and the 1 of them the only participating oral hygienist.

### Quality of communication between HP and rheumatologists

**Fig. 1:**
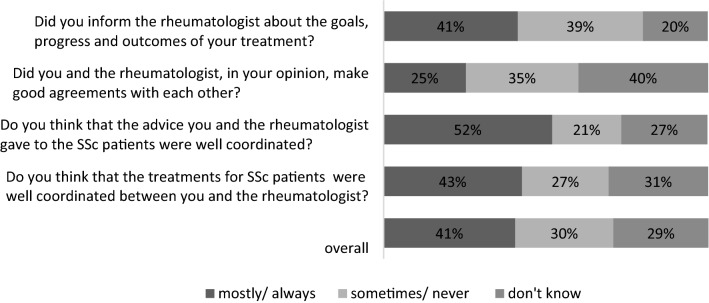
Quality of communication between HPs and rheumatologists, %

Figure 1 illustrates the percentage of participants’ perceptions on the quality of communication. Nearly one-third (29%) of those questioned could not make any statements about the cooperation and/or the quality of communication. Overall, slightly above 40% of the participants had a positive view about the quality of communication. One-quarter of the HPs reported that they are mostly satisfied with the agreements they have with the rheumatologists. Almost 40% of HPs rarely or never inform the rheumatologist about the goals, progress, and outcomes of their treatment.

## Discussion

This cross-sectional survey study revealed that HPs use a broad spectrum of treatment goals (unique ICF codes, *n* = 66) and interventions (*n* = 51). At the HP group level, the ten most common referral reasons and treatment goals were considerably similar. However, analysis at an individual level indicated discrepancies between the self-reported referral reasons provided by the HPs and the reported treatment goals, suggesting insufficient alignment between referral reasons and treatment goals. On the other hand, we demonstrated that HP treatment goals indeed match the most important unmet care needs of SSc patients reported in the literature. Another critical finding was that relatively few HPs communicated with the rheumatologists and only some HPs reported to have agreements with rheumatologists, implying a poor quality of communication between HPs and rheumatologists.

### Missing coherence between referral reasons and HP approach

We found discrepancies between rheumatologists’ referral reasons and the reported HP treatment goals and interventions. HPs report interventions that are not mentioned in the referral reasons such as education, psychosocial interventions, and interventions aimed at social or environmental factors. A possible explanation for this may be insufficiently targeted referrals by rheumatologists. One study among Dutch rheumatologists specialized in SSc, the rheumatologists indicated to be insufficiently aware of the non-pharmacological treatment options [15]. In the absence of available evidence-based guidelines, practice-based evidence recommendations based on consensus could be a good option to share information about existing HP treatment options with referrers and patients. In addition, practice-based or consensus-based non-pharmacological recommendations could also be a good adjunct to the next update of the European League Against Rheumatism (EULAR) recommendations for the treatment of SSc of Kowal-Bielecka et al. [25].

### Good correspondence between treatment goals and unmet care needs and SSc-ICF core set

Our results establish that the reported treatment goals covered the five most important unmet care needs of patients with SSc: fatigue, Raynaud’s phenomenon, joint problems, physical function, and hand function described by Spierings et al. (2019). Our findings complement those of a European study by Willems et al. [26] on the content of HP SSc care identifying fatigue, Raynaud’s phenomenon, and hand function as the most important treatment goals. These results suggest that HPs are indeed able to identify the care needs of patients with SSc. Despite these promising results, written consensus- and evidence-based recommendations need to be established to make the possibilities of HP care more visible for patients with SSc and rheumatologists.

### Large overlap in interventions

Our results reveal that in some cases up to six disciplines indicate that they focus on the same areas of intervention. Due to the quantitative nature of our study, it is unclear whether they actually offer the same interventions or whether they are working with a different focus and intervention strategy. This overlap of the intervention offer could make it difficult for referrers to refer patients with SSc targeted to the best matching HP discipline because the spectrum of interventions offered is large but without clear distinctions. Studies with a qualitative approach could help to further specify the content of the interventions offered and allow referrers to make more targeted referrals to the most appropriate HP disciplines [27].

### Quality communication between HPs and rheumatologists

Our results suggest a suboptimal communication of the HPs with the rheumatologists. Due to transitions of the health care system in the Netherlands, the work setting of HPs delivering care for patients with rheumatic diseases moved from larger hospitals to primary care setting. As a result, possibilities for specialization and multidisciplinary collaboration for HPs in the Netherlands have thinned, thereby reducing direct interaction of HPs with their medical and other HP colleagues. Due to the broad alignment with different target groups in primary care, there is a decrease in specialized HPs for the treatment of rare disorders. This new situation requires new models of care because the complex situation of people with SSc requires specialized care. A digital network, such as ParkinsonNet [28], could be a possible component of such a new care model. ParkinsonNet is a network of more than 3400 specialized health care providers with national coverage in the Netherlands. The model of ParkinsonNet has also been adopted in other countries [29]. Such a network could connect patients and the various health care providers in a targeted manner and thereby increase communication, the quality of multidisciplinary collaboration, and thus the quality of SSc care.

This study had a number of limitations. One limitation was the rather low response rate. We expected that by approaching the HPs through the 650 SSc patients who had participated in the previous survey study, a larger number of HPs would be reached. A possible explanation for the relatively small number of participating HPs is that they, although invited by their patients, subsequently did not participate because they felt insufficiently specialized in SSc. This explanation is supported by the low number of HPs (around 25%) that reported to feel specialized in SSc treatments. The second limitation is the limited use of validated questionnaires, for instance to examine the heterogeneity of interventions. Another limitation might be that our results on referral reasons are based on self-report by HPs, which could lead to recall bias. A content analysis of referral letters would be an option to obtain more reliable information.

## Conclusion

We found a broad spectrum of treatment options offered by Dutch HPs that address the unmet care needs of patients with SSc. An overlap in the content of the care delivered by the various HP disciplines was noted, and the referrals of rheumatologists were not sufficiently aligned with HP treatment goals. The HP offer seems to be inefficiently organized, which may prevent rheumatologists from making targeted referrals. Strategies for better communication between rheumatologists and HPs should be developed and implemented.

## Data Availability

The datasets used and/or analyzed during the current study are available from the corresponding author on reasonable request.

## Appendix

See Table 5.

**Table 5:** Reasons for referral and treatment goals as reported by 79 HPs, expressed in ICF codes

ICF code		Referral reasons *n*	Treatment goals *n*
Body functions			
b1300	Energy level	6	2
b1302	Appetite	1	
b134	Sleep functions		1
b152	Emotional functions	4	4
b1521	Regulation of emotion		1
b1564	Tactile perception	1	
b160	Thought functions	1	1
b240	Sensations associated with hearing and vestibular function		1
b280	Sensation of pain	8	11
b28016	Pain in joints		2
b415	Blood vessel functions		1
b440	Respiration functions	7	3
b4450	Functions of the thoracic respiratory muscles		3
b4550	General physical endurance	1	
b4551	Aerobic capacity	11	25
b4552	Fatigability		1
b510	Ingestion functions	1	1
b5104	Salivation	1	
b5105	Swallowing	2	1
b5150	Transport of food through stomach and intestines	2	
b5152	Absorption of nutrients	1	
b525	Defecation functions		1
b5251	Fecal consistency	1	
b530	Weight maintenance functions	6	2
b5501	Maintenance of body temperature		2
b710	Mobility of joint functions	8	4
b7101	Mobility of several joints		12
b730	Muscle power functions	5	12
b735	Muscle tone functions		3
b760	Control of voluntary movement functions		1
b780	Sensations related to muscles and movement functions		2
b810	Protective functions of the skin		2
b820	Repair functions of the skin	1	
b830	Other functions of the skin		7
Body structures			
s320	Structure of mouth	1	2
s3200	Teeth	1	
s3201	Gums	1	
s430	Structure of respiratory system	1	1
s5	Structures related to the digestive, metabolic and endocrine systems	3	
s710	Structure of head and neck region	1	
s720	Structure of shoulder region	1	
s7201	Joints of shoulder region		1
s7202	Muscles of shoulder region		2
s73011	Wrist joint		1
s7302	Structure of hand	4	
s73021	Joints of hand and fingers		2
s7502	Structure of ankle and foot	3	1
s76000	Cervical vertebral column		1
s76001	Thoracic vertebral column		2
s7701	Joints	2	
s8	Skin and related structures	2	
s8102	Skin of upper extremity	1	
Activities and participation			
d230	Carrying out daily routine	8	2
d2301	Managing daily routine	2	15
d2303	Managing one’s own activity level	7	15
d240	Handling stress and other psychological demands	1	2
d4104	Standing		1
d4105	Bending		1
d415	Maintaining a body position		1
d420	Transferring oneself	1	1
d4301	Carrying in the hands		1
d4401	Grasping		1
d445	Hand and arm use	5	6
d450	Walking	2	5
d455	Moving around	5	6
d460	Moving around in different locations	1	2
d4750	Driving human-powered transportation		2
d520	Caring for body parts		1
d5200	Caring for skin		2
d5201	Caring for teeth		1
d540	Dressing		3
d5701	Managing diet and fitness	1	9
d5702	Maintaining one’s health		1
d6	Domestic life	1	
d6200	Shopping		1
d6300	Preparing simple meals		1
d6505	Taking care of plants, indoors and outdoors		1
d845	Acquiring, keeping, and terminating a job	1	
d8451	Maintaining a job		2
d920	Recreation and leisure		1
d9201	Sports	1	2
Environmental factors			
e110	Products or substances for personal consumption		1
e1100	Food		1
e115	Products and technology for personal use in daily living	1	1
e1151	Assistive products and technology for personal use in daily living		1
e120	Products and technology for personal indoor and outdoor mobility and transportation	2	
e3	Support and relationships	1	

## Abbreviations

SSc: Systemic sclerosis
HPs: Health professionals in rheumatology
STROBE: Strengthening the Reporting of Observational Studies in Epidemiology
ARCH: Arthritis Research and Collaboration Hub
ICF: International Classification of Function and Disability
EULAR: European League Against Rheumatism
CQI: Consumer quality index
